# Absolute Lymphocyte Count Is Not a Suitable Alternative to CD4 Count for Determining Initiation of Antiretroviral Therapy in Fiji

**DOI:** 10.1155/2014/715363

**Published:** 2014-10-27

**Authors:** Dashika A. Balak, Karen Bissell, Christine Roseveare, Sharan Ram, Rachel R. Devi, Stephen M. Graham

**Affiliations:** ^1^Reproductive Health Clinic, Ministry of Health, P.O. Box 30, Suva, Fiji; ^2^International Union Against Tuberculosis and Lung Disease, 75017 Paris, France; ^3^The University of Auckland, Auckland 1020, New Zealand; ^4^Regional Public Health Service, Hutt Valley 5010, New Zealand; ^5^Department of Health Science, College of Medicine, Nursing and Health Sciences, Fiji National University, Suva, Fiji; ^6^Centre for International Child Health, Royal Children's Hospital, University of Melbourne and Murdoch Children's Research Institute, Melbourne, VIC 3052, Australia

## Abstract

*Introduction*. An absolute lymphocyte count is commonly used as an alternative to a CD4 count to determine initiation of antiretroviral therapy for HIV-infected individuals in Fiji when a CD4 count is unavailable. *Methods*. We conducted a retrospective analysis of laboratory results of HIV-infected individuals registered at all HIV clinics in Fiji. *Results*. Paired absolute lymphocyte and CD4 counts were available for 101 HIV-infected individuals, and 96% had a CD4 count of ≤500 cells/mm^3^. Correlation between the counts in individuals was poor (Spearman rank correlation *r* = 0.5). No absolute lymphocyte count could be determined in this population as a suitable surrogate for a CD4 count of either 350 cells/mm^3^ or 500 cells/mm^3^. The currently used absolute lymphocyte count of ≤2300 cells/*μ*L had a positive predictive value of 87% but a negative predictive value of only 17% for a CD4 of ≤350 cells/mm^3^ and if used as a surrogate for a CD4 of ≤500 cells/mm^3^ it would result in all HIV-infected individuals receiving ART including those not yet eligible. Weight, CD4 count, and absolute lymphocyte count increased significantly at 3 months following ART initiation. *Conclusions*. Our findings do not support the use of absolute lymphocyte count to determine antiretroviral therapy initiation in Fiji.

## 1. Introduction

The CD4 count is a measure of immune status in HIV-infected individuals which measures the severity of immunosuppression more accurately than clinical staging of disease. It is therefore included in international (WHO/UNAIDS) and National AIDS Programme guidelines as a threshold marker to indicate the initiation of antiretroviral therapy (ART) [1, 2]. However, an accurate determination of CD4 cell count requires flow cytometry which is often unavailable in resource-limited settings for a variety of reasons [2]. If CD4 testing is not available, WHO guidelines have previously recommended using clinical staging either alone or in combination with an absolute lymphocyte count for determining ART eligibility [3].

In Fiji, a CD4 cell count is recommended to determine eligibility for initiating ART but is often unavailable. An absolute lymphocyte count is therefore used as an alternative, with a count of ≤2300 cells/*μ*L being used to determine eligibility for ART in an HIV-infected individual with clinical stage 1 or 2. However, there has been no evaluation of this practice. Furthermore, Fiji is now implementing the recent World Health Organization (WHO) guidelines which use a higher CD4 count (≤500 cells per mm³) than previously (≤350 cells per mm³) to determine eligibility [1]. The implication of this change for the practice of using an absolute lymphocyte count as an alternative is not known.

This study aimed to describe the CD4 counts and absolute lymphocyte counts in HIV-infected Fijians prior to initiation of ART, to determine the correlation between them in individual patients, and to evaluate the accuracy of a range of threshold values of absolute lymphocyte counts as an alternative to the recommended CD4 count threshold.

## 2. Methods

### 2.1. Study Population and Setting

This was a retrospective study which evaluated clinical records from all HIV-infected individuals in Fiji up until 31 December 2012. Fiji is a low to middle income country with a population of 837,271 (2007 census) [4]. The Ministry of Health administers three major divisions and each division has a Reproductive Health Clinic that provides care for all HIV-infected individuals in Fiji. The first case of HIV infection was diagnosed in Fiji in 1989 and ART was introduced in 2004. Following the diagnosis of HIV infection, assessment for eligibility for ART is undertaken at the Reproductive Health Clinics.

Blood samples were taken at the Reproductive Health Clinics and sent within 6–24 hours to the divisional hospital laboratory for full blood count, including total white blood cell and lymphocyte counts, and to the Fiji Centre for Communicable Disease Control for CD4 cell count (Alere Pima Analyser). CD4 testing became available in Fiji in 2010 but has been unavailable since early 2012 due to stock-out of laboratory reagents.

At the time of initial clinical and laboratory assessment of the HIV-infected individuals included in the study, the guidelines in Fiji recommended that ART be initiated in HIV-infected individuals categorised as WHO clinical staging 3 or 4 and in those with a CD4 cell count of ≤350 cells/mm^3^ regardless of the clinical stage. When CD4 count was not available, the senior HIV clinician (DAB) used an absolute lymphocyte count value of 2300 cells/*μ*L as a surrogate for a CD4 cell count of 350 cells/mm^3^ on the basis of studies from other settings. The national guidelines have recently been revised to be consistent with the new WHO guidelines that now recommend initiation of ART in HIV-infected individuals that are categorized as WHO clinical stage 1 or 2 with a CD4 cell count of ≤500 cells/mm^3^ [3, 5].

### 2.2. Data Collection and Analysis

Data were obtained from individual patient folders from all three Reproductive Health Clinics. Only those with complete data available for results of both absolute lymphocyte count and CD4 count for baseline and follow-up were included in the analysis. Variables included age, gender, ethnicity, weight, the WHO clinical staging, and laboratory markers that included absolute lymphocyte count, haemoglobin, and CD4 cell count taken before and 3 months after initiation of ART.

Data were collected and double-entered directly into an electronic data collection sheet (Epi Data 3.1). Data were compared for discordance and corrections were made by verification with the patient folders to produce a finalized data set. Data were analysed using Epi Data Analysis 2.1 software. The Spearman rank correlation was used to compare absolute lymphocyte count with CD4 counts. Positive predictive value, negative predictive value, and sensitivity and specificity of varying ALC cut-offs were computed for CD4 counts ≤350 cells/mm³ and ≤500 cells/mm³. The paired *t*-test was used to compare the differences in clinical markers in response to ART.

### 2.3. Ethics

Ethics approval for the study was obtained from the Ethics Advisory Group of the International Union Against Tuberculosis and Lung Disease, the Fiji National Health Research Committee, and National Research Ethics Review Committee.

## 3. Results

**Figure 1:**
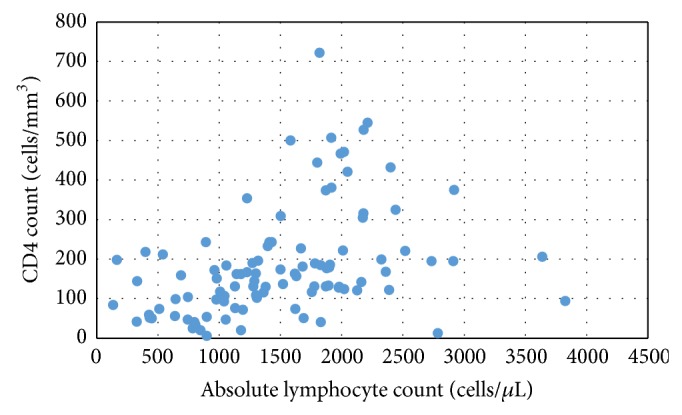
Distribution of absolute lymphocyte count and CD4 cell count in HIV-infected individuals before initiation of antiretroviral therapy.

The data from 101 HIV-infected patients were available and were included in the study. The mean age of the patients was 34 years (range: 18 to 61 years) and 50% were males. Prior to initiation of ART, the median absolute lymphocyte count was 1429 cells/*μ*L (range: 135 to 3825 cells/*μ*L) and the median CD4 cell count was 157 cells/mm³ (range: 6 to 722 cells/mm³). The correlation between absolute lymphocyte count and CD4 cell count was weak (*r* = 0.497) and this is illustrated in Figure 1.  Table 1 shows the frequency distribution and corresponding WHO clinical stage of patients using various CD4 cut-offs that have been applied in recommendations for the initiation of ART. The majority of the patients had a CD4 count of ≤200 cells/mm³ at the time of initial assessment, and 86% had a CD4 count of ≤350 cells/mm³ which would indicate eligibility for ART irrespective of clinical staging applying the guidelines in use at the time.

Of the 101 HIV-infected individuals, 88 (87%) had an absolute lymphocyte count of <2300 cells/*μ*L and 59 (58%) were either clinical stage 1 or 2 and so required CD4 count data to determine eligibility for ART. However, the majority of the 59 patients assessed as clinical stage 1 or 2 had CD4 counts of ≤350 cells/mm³ or of ≤500 cells/mm³, 46 (78%) and 55 (93%), respectively. Of the 46 clinical stage 1 or 2 patients eligible to initiate ART on the basis of a CD4 count of ≤350 cells/mm³, 6 (13%) had absolute lymphocyte counts of greater than 2300 cells/*μ*L and so would have been wrongly denied initiation of ART if absolute lymphocyte count alone was used. On the other hand, all four (4%) HIV-infected patients that had a CD4 count of >500 cells/mm³ and were clinical stage 1, and so ineligible for ART, had an absolute lymphocyte count of less than 2300 cells/*μ*L.

Table 2 lists the calculated positive predictive values, negative predictive values, and sensitivities and specificities for a range of different absolute lymphocyte counts cut-offs that might be used as a surrogate for a CD4 cell count of ≤350 cells/mm³. As expected, increasing the absolute lymphocyte count cut-off increased the sensitivity of the test for determining those with a CD4 count of ≤350 cells/mm³, but with the cost of reduction in specificity. The sensitivity of the absolute lymphocyte count in use in Fiji that has been used (i.e., <2300 cells/*μ*L) was 88.5% for a CD4 of ≤350 cells/mm³ in all patients, reflecting that the majority of patients had low numbers of both counts (Figure 1). However, the specificity was only 13.5% as the majority (12 of 14) of patients with a CD4 of >350 cells/mm³ had an absolute lymphocyte count level of <2300 cells/*μ*L (Figure 1). Absolute lymphocyte counts for a cut-off of ≤500 cells/mm³ showed similar sensitivity to that found for a CD4 count of ≤350 cells/mm³ (data not shown). An absolute lymphocyte count of 2300 cells/*μ*L had a sensitivity of 87.6% for a CD4 count of 500 cells/mm³ but a specificity of 0% because all four patients with a CD4 of >500 cells/mm³ had an absolute lymphocyte count level of <2300 cells/*μ*L.

The response to ART for weight and laboratory markers after 3 months of initiation is shown in Table 3. There was significant weight gain and increase in ALC and CD4 counts after 3 months of ART but no change in haemoglobin values.

## 4. Discussion

This study provides original data from Fiji of clinical staging, CD4 counts, and absolute lymphocyte counts in HIV-infected individuals at the time of assessment for initiation of ART. Of the 101 individuals, 42% were indicated to receive ART on the basis of clinical staging alone. Of the remaining individuals of clinical stage 1 or 2 that required a CD4 count of ≤350 cells/mm³ to determine eligibility, as per recommendations at the time, the use of an absolute lymphocyte count of 2300 cells/*μ*L as a surrogate cut-off would have resulted in incorrect management in 10 (17%) of 59 individuals: 6 eligible for ART that would not receive ART and an additional 4 not yet eligible for ART that would have started ART. With adoption of the recent recommendations of using a CD4 count cut-off of 500 cells/mm³ to determine eligibility, using an absolute lymphocyte count of 2300 cells/*μ*L as a surrogate cut-off would have resulted in all individuals receiving ART including the 4 individuals with clinical stage 1 disease that had CD4 counts >500 cells/mm³.

These findings are important and currently relevant for two main reasons. Firstly, in line with WHO recommendations, the Fiji national guidelines have recently increased the CD4 cell count to initiate ART to ≤500 cells/mm^3^ from the previous ≤350 cells/mm^3^ [1, 2]. This will increase the number of HIV-infected individuals that are eligible to receive ART. We found that almost all (96%) HIV-infected individuals had a CD4 count at the time of assessment that was ≤500 cells/mm³. Secondly, there is only one PIMA CD4 machine for CD4 testing in Fiji and the CD4 test continues to be frequently unavailable due to the lack of laboratory reagents for testing. However, using an absolute lymphocyte count as a surrogate would have resulted in all individuals receiving ART. Due to the low absolute lymphocyte counts and CD4 counts in the majority of this population and a weak correlation between counts within individuals, it was difficult to determine an optimal cut-off that provides an acceptable balance between sensitivity and specificity. For example, if a low absolute lymphocyte count level with a high specificity was used, such as <1500 cells/*μ*L, then nearly half of the HIV-infected individuals eligible for ART by CD4 criteria would not receive treatment.

There are a number of study limitations. An important limitation is the small number of HIV-infected individuals with complete data available for retrospective analysis. This study population of 101 individuals represented a convenience sample that was included on the basis of having complete test results available, when CD4 testing was only available for a limited time from 2010. It is not certain therefore how representative this group might be for the wider population of HIV-infected individuals in Fiji. Further, clinical staging data were not collected prospectively and so it is difficult to validate accuracy of the clinical categorisation.

It is also not possible to know the wider relevance of these findings to other populations. There have been no previous studies from the Oceania region. Previous similar studies from a range of settings including early studies from the United States, England, and South Africa [6–9] and more recent studies from low-resource settings such as India and Nigeria [10–13] have found variable results and have interpreted similar results differently. There are many factors that are likely to influence the results of absolute lymphocyte counts and CD4 counts in different populations apart from the effect of HIV infection, which include prevalence of other infections as well as other biological and genetic factors. Our study could not determine the potential effect of any of these factors. Our study also did not evaluate pregnant women and children separately. These groups have different guidelines for ART initiation which do not rely on CD4 counts [3] and previous studies have shown that ALC would not be a substitute for CD4 count in these populations [14, 15].

We did find that absolute lymphocyte count increased in response to 3 months of ART as did CD4 count and weight. However, we did not determine whether ALC could be a suitable alternative to CD4 to monitor response after 3 months of ART or would provide additional benefit over clinical improvement and weight gain.

In conclusion, the results of this study would not support the continued use of absolute lymphocyte count to determine ART initiation in HIV-infected individuals in Fiji. Rather, when CD4 count is unavailable, the introduction of a recommendation to initiate all HIV-infected individuals on ART should be considered.

## Figures and Tables

**Table 1:** Frequency distribution of the various CD4 cut-off values and corresponding WHO clinical stage for the initiation of antiretroviral therapy (*n* = 101).

CD4 cut-off values (cells/mm³)	Number (% of total)	WHO clinical stage *N* (% of total)			
		1	2	3	4
≤200	73 (72)∗	15 (48)	19 (68)	22 (92)	17 (94)
≤350	87 (86)	19 (61)	27 (96)	24 (100)	17 (94)
≤500	97 (96)	27 (87)	28 (100)	24 (100)	18 (100)
>500	4 (4)	4 (13)	0	0	0

Total	101	31	28	24	18

^*^Note that the numbers and percentages in each row are cumulative up to the ≤500 cells/mm^3^ cut-off.

**Table 2:** Calculated positive predictive values (PPV), negative predictive values (NPV), and sensitivities and specificities of absolute lymphocyte counts (ALC) for CD4 count ≤350 in all the paired counts (*n* = 101).

ALC (cells/*µ*L)	PPV (%)	NPV (%)	Sensitivity (%)	Specificity (%)
<1500	98.0	26.0	57.5	92.9
<1600	96.4	26.1	60.9	85.7
<1700	96.7	30.0	67.8	85.7
<1800	96.9	32.4	71.3	85.7
<1900	93.2	32.1	78.2	64.3
<2000	89.9	27.3	81.6	42.9
<2100	88.0	22.2	83.9	28.6
<2200	87.5	23.1	88.5	21.4
<2300	86.5	16.7	88.5	13.5
<2400	87.0	22.2	92.0	14.3
<2500	86.2	14.3	93.1	7.1

**Table 3:** Comparison of laboratory markers and weight in HIV-infected individuals before and 3 months after initiation of antiretroviral therapy (ART).

Variables	Mean values		*P* value
	Before ART initiation	3 months after ART initiation	
Absolute lymphocyte count (cells/*µ*L)	1408.59	1586.43	<0.01
CD4 count (cells/mm³)	137.76	230.09	<0.001
Haemoglobin (g/dL)	12.06	12.28	0.18
Weight (kg)	68.27	71.64	<0.001
